# Comparison between intravitreal ranibizumab injection and posterior subtenon triamcinolone acetonide injection at time of cataract surgery for prevention of progression of diabetic macular edema

**DOI:** 10.1186/s12886-022-02625-2

**Published:** 2022-12-16

**Authors:** Mahmoud Mohammed Ahmed Ali Khalil, Hosam Othman Mansour, Ahmed Mohamed Raafat Tawfik, Ahmed Gomaa Elmahdy

**Affiliations:** 1 Department of Ophthalmology, Al Azhar University, Cairo, Egypt; 2 Magrabi Eye Hospital Tanta, Tanta, Egypt; 3 Department of Ophthalmology, Faculty of Medicine, Al Azhar University, Damietta branch, New Damietta, Egypt

**Keywords:** Subtenon Triamcinolone acetonide, Intravitreal ranibizumab, Phacoemulsification, Diabetic macular edema

## Abstract

**Background:**

The goal of this work is to assess progression of diabetic macular edema (DME) following intravitreal ranibizumab injection compared to subtenon triamcinolone acetonide injection at cataract operation.

**Methods:**

Retrospective analysis of 73 eyes of 65 participant with DME, with central macular thickness (CMT) ≥ 300 μm. The included eyes were separated into three groups; phacoemulsification with intravitreal Ranibizumab injection group, phacoemulsification with subtenon Triamcinolone acetonide injection group and phacoemulsification only group. Main measures involved best corrected visual acuity (BCVA) one week, one month and three months post-operative. The CMT was compared preoperative and postoperative (one and three months).

**Results:**

After 1 month of operation, there was a statistical substantial distinction in the median of CMT between ranibizumab & control group (*p* < 0.001), between subtenon TA & control group (*p* < 0.001) and in ranibizumab and subtenon TA group (*p* = 0.023). After 3 months, the variance between ranibizumab & control group was considerable (*p* < 0.0001) and the variance between subtenon TA & control group was considerable (*p* = 0.030).

**Conclusions:**

Combined phacoemulsification with intravitreal injections of ranibizumab or subtenon triamcinolone acetonide may prevent further progression in CMT in individuals with DME following cataract operation.

## Introduction

Diabetic macular edema (DME) is the main cause for visual loss in diabetic patients [1]. Diabetes is proven to rise DME occurrence by a twenty two percent effect on optical coherence tomography (OCT) following cataract operation [2]. Many studies have shown that posterior subtenon or intravitreal injection triamcinolone acetonide to be beneficial for decreasing central macular thickness in patients with DME [3]. Effect of anti-VEGF drugs in combination with cataract surgery in preventing the progression of DME has been confirmed by several reports [4–7]. In this work, a comparison between intravitreal ranibizumab and posterior subtenon triamcinolone acetonide injection at time of cataract operation was evaluated together with a control group for the prevention of progression of DME in diabetic patients.

## Patients and methods

A multicentric retrospective analysis of the medical records of 73 eyes for 65 diabetic patients underwent phacoemulsification surgery. The study was conducted at ophthalmology department, Al-Azhar University Hospitals, Egypt in the period from January 2019 till September 2020. The included eyes were divided into three groups;

- Group 1: included 21 eyes undergone combined phacoemulsification and intravitreal ranibizumab injection, 0.1 mL of a solution containing 0.5 mg of ranibizumab (Lucentis).
- Group 2: included 23 eyes undergone combined phacoemulsification and posterior subtenon triamcinolone acetonide injection.
- Group 3: included 29 eyes as a control group with phacoemulsification only.

### Inclusion criteria

Diabetic patients who had a cataract associated with non-tractional DME and never received any type of treatment for this DME (neither injections nor laser photocoagulation). Tight glycemic control according to HBA1C was mandatory preoperatively.

### Exclusion criteria

Patients with dense cataract obscured central macular thickness calculation, vitreo retinal tractions, chronic uveitis and pathology of the posterior segment other than diabetic retinopathy. Complicated cataract surgery (posterior capsule tears, nuclear drop, iris damage, corneal edema or postoperative inflammation).

### Pre-operative evaluation

All patients were subjected to complete ocular examinations; best corrected visual acuity measured in log MAR scale, slit lamp biomicroscopy of anterior and posterior segments to assess the grade of diabetic retinopathy and diabetic maculopathy under fully dilated pupil.

OCT was done to measure CMT; 6 radial scans focused on the fovea yielding a total of 600 samples from 6 radial scans. The OCT machine used in this study was Topcon DRI OCT triton with automatic measurement of central macular thickness (from ILM to RPE). 3 readings were obtained to verify the measurements.

### Surgical technique

Sterilization using povidone iodine 5% to sterilize the ocular surface before surgery. A single surgeon carried out phacoemulsification under local anesthesia. A 2.4 mm keratome and side port with angled MVR 20 gauge were used to make a clear incision in the cornea. A continuous curvilinear capsulorhexis of about 6 mm was fashioned after anterior chamber has been filled with an ophthalmic viscoelastic gel. Nucleus hydrodelineation and dissection. Stop and chop phacoemulsification technique was done. The irrigation aspiration of cortex was then carried out bimanually. A foldable acrylic intraocular lens was injected in the capsular bag. A viscoelastic wash by irrigation aspiration. Hydration of the main and side corneal wound.

Intravitreal ranibizumab or subtenon triamcinolone acetonide injection was done at the end of cataract operation.

#### Subtenon triamcinolone

Incision of the bulbar conjunctiva 8 mm from the limbus at the infero-temporal quadrant, then the Tenon’s capsule is dissected through the wound inferior and posterior to the globe, then 1 ml of 40 mg/ml of triamcinolone acetonide was injected using viscoelastic gauge or blunt 19 gauge infusion cannula and insulin syringe followed by massaging and compression on the conjunctival wound, finally injection of garamycin, dexamethasone subconjunctival and eye patch was used.

#### Intravitreal ranibizumab

0.1 mL of a solution containing 0.5 mg of ranibizumab (Lucentis) was injected 3–3.5 mm from the limbus into the mid vitreous.

Postoperatively, topical antibiotic and steroid drops are similarly prescribed in the three groups.

Main measures involved best corrected visual acuity (BCVA) one week, one month and three months post-operative. OCT was done assessing the CMT (one and three months). Based on OCT, pseudophakic CME is characterized by central macular edema pattern, increased central retinal thickness/retinal volume ratio, increased foveal thickness of ONL/HL and cysts in INL, while in DME there are higher retinal volume, diffuse or focal thickening with preserved foveal contour, high ONL/INL thickness ratio in the parafoveal area, microaneurysms, hard exudates, hyperreflective foci, cysts in INL and other layers and subretinal fluid, if anything was debatable then FFA was done with appearance of hot disc in PCME.

In in the first 3 months, no added injection was done, after the 3 months PRN protocol was applied for all patients with residual or recurrent DME.

### Statistical analysis

Statistical package for social sciences, version 20.0 (SPSS Inc., Chicago, Illinois, USA) was used to analyze the data. Quantitative data were expressed as mean ± standard deviation (SD). Qualitative data were expressed as frequency and percentage. The following tests were done; independent-samples t-test, one-way analysis of variance (ANOVA), post hoc test was used. *P*-value < 0.05 was considered significant.

## Results

This study included 73 eyes from 65 patients. Age of studied patients ranged 35- 70 year. The majority of patients were females (65.8%). 61 patients had Type 2 DM and 12 patients had type 1 DM (Table 1).

**Table 1:** Studied patients’ distribution regarding age, sex and type of diabetes mellitus

**Demographic data**	**Total (*n* = 65)**
**Age (years)**	
Range	35–70
Mean ± SD	54.08 ± 10.27
**Sex**	
Male	25(38.5%)
Female	40 (61.5%)
**Diabetes mellitus**	
Type I	12 (18.5%)
Type II	53 (81.5%)

**Fig. 1:**
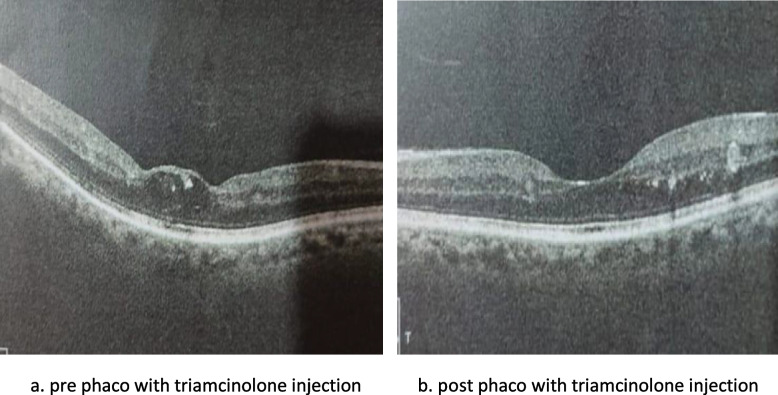
Subtenon triamcinolone acetonide injection: **a** preoperative **b** after 1 month **c** after 3 months

**Fig. 2:**
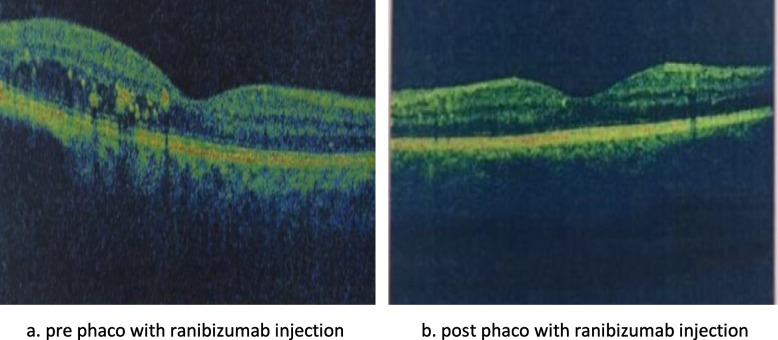
Intravitreal ranibizumab injection: **a** Preoperative **b** after 1 month **c** after 3 months

**Fig. 3:**
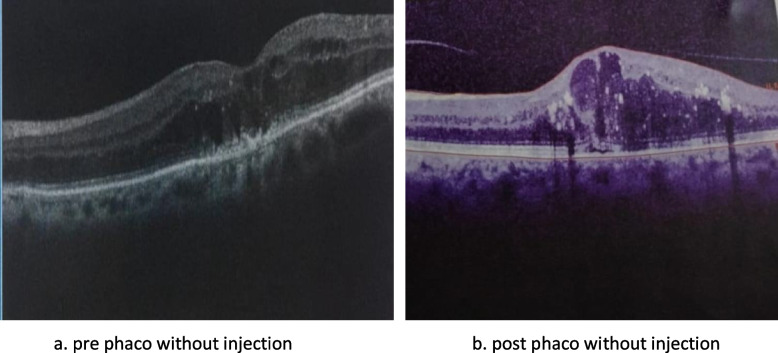
Phacoemulsification without injection: **a** Preoperative **b** after 1 month **c** after 3 months

No significant difference between groups considering baseline CMT (Figs. 1a, 2a, 3a). One month postoperative, mean of CMT decreased in both Ranibizumab and subtenon Triamcinolone acetonide groups (280.7 and 310.1 μm, respectively) while increased in control group 437.1 μm (Figs. 1b, 2b, 3b).

**Fig. 4:**
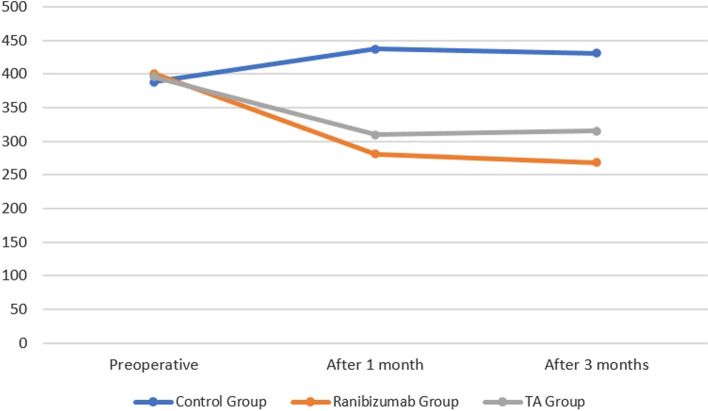
Bar chart between groups regarding CMT

Three months postoperative, mean of CMT decreased in both Ranibizumab & control groups comparing to 1 month (268.13& 431.03 respectively) while increased in subtenon Triamcinolone acetonide group (315.53) (Figs. 1c, 2c, 3c). A significance between groups recorded according to CMT after 1 month and after 3 months (*p* < 0.001) (Fig. 4, Table 2).

**Table 2:** Comparison between groups according to CMT

**CMT**	**Ranibizumab Group *(n* = *21 eyes)***	**Subtenon Triamcinolone acetonide Group *(n* = *23 eyes)***	**Control Group *(n* = *29 eyes)***	**ANOVA**	***p*-value**	**Post Hoc test**
	Mean ± SD	Mean ± SD	Mean ± SD			
Preoperative	400.9 ± 87.69	395.8 ± 105.51	388.6 ± 48.55	0.975	0.751	
After 1 month	280.7 ± 25.94	310.1 ± 42.8	437.1 ± 87.93	10.308	< 0.001**	P1 < 0.001**
						P2 < 0.001**
						P3 = 0.023*
After 3 months	268.13 ± 32.94	315.53 ± 55	431.03 ± 89.66	13.536	< 0.001**	P1 < 0.001**
						P2 < 0.001**
						P3 = 0.009*

Post HOC: p1; Ranibizumab Group compared to control group, P2; Subtenon Triamcinolone acetate Group compared to control group, P3; Ranibizumab Group compared to Subtenon Triamcinolone acetate Group

*p*-value > 0.05 NS; *p-value < 0.05 S; **p-value < 0.001 HS

**Fig. 5:**
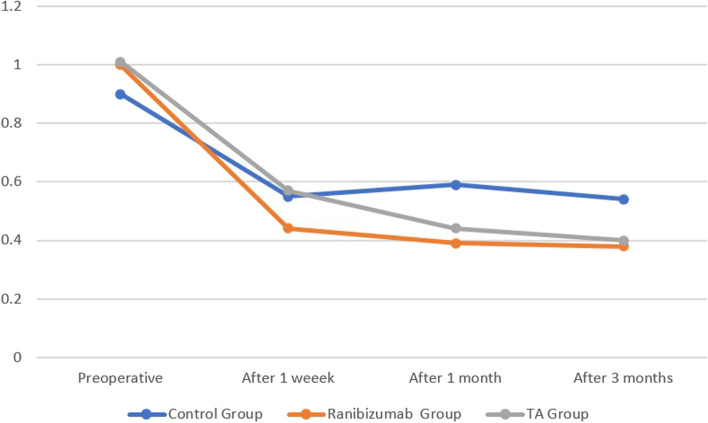
Bar chart between groups regarding VA

No significant difference between groups considering baseline VA in logMAR. After 1 week of surgery, mean of VA decreased in all groups (0.55, 0.44 and 0.57, respectively) with significance between Ranibizumab & control (*p* = 0.14) and between Ranibizumab & subtenon Triamcinolone acetonide groups (*p* = 0.034). After 1 month of surgery, mean of VA decreased more in both Ranibizumab and subtenon Triamcinolone acetonide groups comparing to 1 week (0.39 and 0.44, respectively) while increased in control group 0.59 with significance between each of Ranibizumab and subtenon Triamcinolone acetonide groups and control group (*p* < 0.001 and 0.012, respectively). Mean of VA decreased after 3 months of surgery comparing to 1 month in all groups (0.54, 0.38 and 0.4, respectively) with significance between each of Ranibizumab and subtenon Triamcinolone acetonide groups and control group (*p* < 0.001 and 0.030, respectively) (Fig. 5, Table 3).

**Table 3:** Compare between groups regarding V.A in logMAR

**V.A**	**Ranibizumab Group (*n*** **= 21 eyes)**	**Subtenon Trimcinolone acetonide Group (*n*** **= 23 eyes)**	**Control Group (*n*** **= 29 eyes)**	**ANOVA**	***p*-value**	**Post Hoc test**
	Mean ± SD	Mean ± SD	Mean ± SD			
Preoperative	1.0 ± 0.06	1.01 ± 0.19	0.9 ± 0.09	1.471	0.240	
After 1 week	0.44 ± 0.15	0.57 ± 0.09	0.55 ± 0.17	4.07	< 0.001**	P1 = 0.014* P2 = 195 P3 = 0.034*
After 1 month	0.39 ± 0.16	0.44 ± 0.22	0.59 ± 0.15	4.995	< 0.001**	P1 < 0.001**
						P2 = 0.012*
						P3 = 0.152
After 3 months	0.38 ± 0.16	0.4 ± 0.18	0.54 ± 0.17	3.754	< 0.001**	P1 < 0.001**
						P2 = 0.030*
						P3 = 0.144

F-One Way Analysis of Variance

Post HOC: p1; Ranibizumab Group compared to control group, P2; Subtenon Triamcinolone acetate Group compared to control group, P3; Ranibizumab Group compared toSubtenon Triamcinolone acetonide Group

*p*-value > 0.05 NS; *p-value < 0.05 S; **p-value < 0.001 HS

### Regarding postoperative complications

-Infection: no cases of postoperative infection
-Subconjunctival hemorrhage: in control group, there were no cases, in ranibizumab group only 5 cases while in subtenon TA group there were 8 cases
-Elevated IOP postoperatively, in control group there was only one case in the first day, in ranibizumab group there were 2 cases in the first day while in subtenon TA group, there were 4 cases 2 weeks postoperatively. All cases with elevated IOP were managed with just topical Beta-blocker which stopped once controlled

## Discussion

DME is the most common reason of poor visual outcomes in diabetic patients after cataract surgery. It has been shown that level of aqueous VEGF was significantly positively correlated with a clinically meaningful change in CMT in diabetic patients 1 month following cataract surgery. Of note, the disease severity is also correlated with VEGF level preoperatively [8].

The risk of ME was associated with preoperative grade of retinopathy; the risk of DME in the 1st year postoperatively was 1.0% (no DR preoperatively), 5.4% (mild non‑PDR [NPDR]), 10.0% (moderate NPDR), 13.1% (severe NPDR), and 4.9% (PDR) [9].

We thought that controlling this VEGF increase would play an important role in preventing postoperative increase in CMT, hence improving visual outcome of the patients after cataract surgery. This work showed that intravitreal ranibizumab injection at the time of cataract surgery caused significant decrease in macular thickness as compared to the other groups.

Tatsumi et al. [10] evaluated intravitreal triamcinolone acetonide injection during cataract operation in comparison with subtenon triamcinolone acetonide injection for DME patients. Their findings have shown that the BCVA was markedly increased at three and six months after injections. Other treatments were necessary for 13 eyes in the intravitreal triamcinolone acetonide injection group and 21 eyes in the subtenon triamcinolone acetonide injection group (*p* < 0.05). One case was subjected to intravitreal triamcinolone acetonide injection and selective laser trabeculoplasty was done due to intraocular pressure rise.

In our work the CMT was significantly decreased in the subtenon triamcinolone group as compared to the control group, which was directly reflected on the improvement in visual acuity.

Patel et al. [11] analyzed VEGF values in seven diabetic patients post cataract surgery. On first day after surgery there was a 10-fold rise, and VEGF levels were significantly reduced (2.5-fold) by the end of the first month.

In 2014, Mohamed K, investigated alterations in CMT in diabetics and non-diabetics after uncomplicated phacoemulsification and their visual outcome. His results have shown that in non-diabetic patients, central macular thickness after surgery rises in a small percentage but never reaches postoperative cystoid macular edema, but in diabetics, CMT raised more than non-diabetics and in patients with diabetic maculopathy before surgery more specifically [12].

Meta-analysis was done to examine effect of intravitreal ranibizumab injection at time of cataract surgery in diabetic patients with diabetic-retinopathy (DR). Six studies have been found that describe a total of 283 eyes. The findings from the meta-analysis found that a best corrected vision assessed at one month and three months after cataract operation in the intravitreal ranibizumab injection group was notably better than in the control groups (*P* < 0.00001 and *P* = 0.01), but at 6 months, the best corrected vision did not vary significantly between the two groups (*P* = 0.24). Furthermore, the macular central thickness was considerably less in the intravitreal ranibizumab injection groups at 1, 3 and 6 months postoperatively than the control groups (*P* = 0.01, *P* = 0.0004 and *P* = 0.01). In the control group, DR and maculopathy were more evident at 6 months postoperatively than in the intravitreal ranibizumab injection group (*P* = 0.0001 and *P* < 0.0001, respectively). Also, it showed that cataract operation in combination with intravitreal ranibizumab injection seemed to be a good short-term therapy for patients with coexistent DR (up to 6 months) [13].

Chen et al. [14] revealed a significant visual enhancement and decreasing central macular thickness after intravitreal ranibizumab injection in fifteen patients during cataract surgery.

In our work the mean of CMT was decreased significantly in both ranibizumab group and subtenon TA group in comparison with control group. Comparing ranibiazumab group with subtenon TA group, the CMT was decreased significantly in ranibizumab group. These results indicate that both ranibizumab and TA improved postoperative CMT with relatively better significant results in ranibizumab group.

Limitations of the study were small sample size and its retrospective design. Also, staging of diabetic retinopathy and correlation of this staging with the results weren’t done.

## Conclusion

In diabetic patients with DME, Combined phacoemulsification with intravitreal injection of ranibizumab or subtenon triamcinolone acetonide may decrease the CMT and improve the best corrected visual acuity with relatively better significant results in the intravitreal ranibizumab injection after surgery.

## Data Availability

The datasets used and analyzed during the current study are available from the corresponding author on reasonable request.
